# Acute success and safety of the second-generation PVAC GOLD phased RF ablation catheter for atrial fibrillation

**DOI:** 10.1007/s10840-020-00728-8

**Published:** 2020-04-06

**Authors:** M. N. Klaver, L. I. S. Wintgens, M. C. E. F. Wijffels, J. C. Balt, V. F. van Dijk, A. Alipour, S. M. Chaldoupi, R. Derksen, L. V. A. Boersma

**Affiliations:** 1 Department of Cardiology, St. Antonius Hospital, Koekoekslaan 1, 3435CM Nieuwegein, The Netherlands; 2 Department of Cardiology, Amsterdam University Medical Centres, location Academic Medical Centre, Amsterdam, The Netherlands; 3 Department of Cardiology, Rivierenland Hospital, Tiel, The Netherlands; 4 Department of Cardiology, Maastricht University Medical Centre, Maastricht, The Netherlands; 5 Department of Cardiology, Rijnstate Hospital, Arnhem, The Netherlands

**Keywords:** Atrial fibrillation, Catheter ablation, Phased radiofrequency, PVAC GOLD, Pulmonary vein isolation, Safety

## Abstract

**Purpose:**

The second-generation multi-electrode catheter, pulmonary vein ablation catheter (PVAC) GOLD, was designed to improve the delivery of phased radiofrequency energy and reduce procedure times using a ‘single-shot’ approach for pulmonary vein isolation (PVI), while retaining efficacy and safety. This large registry presents acute success rates and safety outcomes in a daily practice setting.

**Methods:**

A total of 1017 patients undergoing first-time ablation for atrial fibrillation (AF) using PVAC GOLD were included, 644 patients with paroxysmal AF and 373 patients with non-paroxysmal AF, divided into 175 patients receiving PVI only and 198 patients receiving PVI with additional substrate modification.

**Results:**

High and comparable percentages of successful PVI could be achieved in all groups (98%, 95% and 99%; *p* = 0.108). The median total procedure time for all groups was 90 min [70–100]. As expected, the total procedure, ablation and fluoroscopy time were significantly longer in the PVI + substrate modification group compared with the PVI-only cases (all *p* < 0.001), but not between the PVI-only groups (*p* = 0.306, *p* = 0.088, *p* = 0.233, respectively). A total of 44 complications were observed in 43 patients (4.2%). Major complications were seen in 19 patients (1.87%) and non-major procedure–related complications were seen in 25 patients (2.46%). Complications leaving permanent sequelae were rare and occurred in only four patients (0.39%). Complications did not differ between groups (*p* = 0.199, *p* = 0.438, *p* = 0.240 and *p* = 0.465 respectively).

**Conclusion:**

PVAC GOLD performs successful PVI, while reducing procedure times and retaining safety for paroxysmal, persistent and long-standing persistent AF. Safety was unaffected by additional substrate modification.

**Electronic supplementary material:**

The online version of this article (10.1007/s10840-020-00728-8) contains supplementary material, which is available to authorized users.

## Introduction

Catheter ablation of atrial fibrillation (AF) is a well-established treatment option for patients with symptomatic, drug refractory A [1]. Furthermore, the incidence of AF and indication for pulmonary vein isolation (PVI) continue to rise with the ageing population [1, 2]. Conventional point-by-point procedures require high operator skills and are often lengthy. This leads to an increasing demand for operating rooms and operator time. The multi-electrode phased radiofrequency pulmonary vein ablation catheter (PVAC) was designed to allow circumferential ablation of the pulmonary veins (PV), using a ‘single-shot’ approach and reduce procedure times, while retaining efficacy and safety. Although successful in achieving these targets, concerns about asymptomatic cerebral embolism (ACE) led to the design of the second-generation multi-electrode catheter, PVAC GOLD, to mitigate emboli and improve the delivery of phased RF energy [3, 4]. Additional targets can be ablated using the multi-array septal catheter (MASC) and multi-array ablation catheter (MAAC) which may include the elimination of non-pulmonary vein triggers and arrhythmogenic substrates in the atria. Limited data is available evaluating the performance and safety of the second-generation PVAC GOLD to achieve PVI and the safety of additional substrate modification using multi-electrode phased RF ablation. This large single-centre registry was designed to fulfil this knowledge gap and present data in an all-comers daily practice setting.

## Methods

### Study design and population

The PVAC GOLD registry is a retrospective, single-centre registry of all consecutive patients undergoing a first-time ablation procedure for AF using the second-generation PVAC GOLD phased RF ablation catheter. Patients with paroxysmal, persistent and long-standing persistent AF treated between May 2013 (introduction) and December 2016 with the PVAC GOLD phased RF ablation system were enrolled in the study. No exclusion criteria were used to depict an everyday practice population. Patients enrolled in a different clinical trial or who underwent a concomitant left atrial appendage closure during the same procedure were excluded from analyses to avoid publication bias. The study was conducted in compliance with Good Clinical Practice Guidelines and was approved by the Ethics Committee and local institutional review board.

All patients had documented symptomatic AF and were classified according to the 2017 expert consensus statement as paroxysmal AF (terminates spontaneously or with intervention within 7 days of onset), persistent AF (continuous AF that is sustained beyond 7 days) or long-standing persistent AF (continuous AF of greater than 12-month duration) [2]. Patients were allocated to one of three groups: firstly, patients with paroxysmal AF, who underwent ablation of the PVs only (‘PAF-PVI’). Patients with persistent or long-standing persistent AF were divided into two groups: those who underwent ablation of the PVs only (‘PersAF-PVI’) and patients who underwent ablation of the PVs with additional ablation of complex fractionated atrial electrograms (CFAE) in the left atrium (‘PersAF-PVI + CFAE’).

### Ablation procedure

**Fig. 1:**
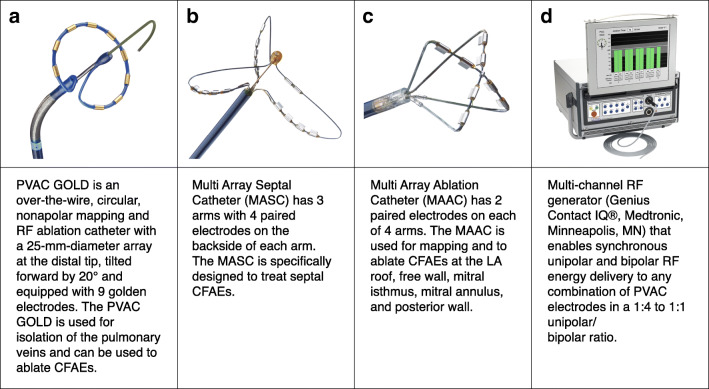
Ablation tools for pulmonary vein isolation and substrate modification. RF, radiofrequancy; CFAE, complex fractionated atrial electrogram; LA, left atrium

The ablation procedure was performed following a standardized ablation protocol as previously described [3, 5]. The procedures were performed under continued oral anticoagulation with vitamin K antagonists (VKA) (target international normalized ratio between 2.0 and 3.0) or non–vitamin K oral anticoagulants (NOAC). Ablation procedures were performed under conscious sedation with intravenous diazepam and fentanyl or, in selected cases, general anaesthesia. Ultrasound-guided vascular access or transseptal puncture (TSP) was only performed in a small number of selected cases. After a TSP, a 10-Fr or larger sheath was inserted into the left atrium. Selective contrast injections were used to visualize the anatomy and pulmonary vein ostia. Normal PV anatomy was defined as 4 separate PV ostia. All patients were then given an intravenous heparin bolus of 7500–10.000 IU based on weight. In case a left atrial dwell time of more than 1 h was needed, a second bolus of 5000 IU was administered. After selective pulmonary vein angiography, the PVAC GOLD catheter was introduced and positioned in the PV antrum using an over-the-wire technique and PV potentials were recorded using the PVAC GOLD catheter. RF energy was applied using predominantly 4:1 and 2:1 setting and occasional 1:1 bipolar/unipolar ratio at the discretion of the physician. After electrical isolation of the PVs, patients in the PersAF-PVI + CFAE group underwent additional ablations in the left atrium using PVAC GOLD or the MASC and MAAC. Septal ablation with the MASC and left atrial wall ablation with the MAAC was performed using a 1:1 bipolar/unipolar ratio or 4:1 bipolar/unipolar ratio at the posterior wall. After CFAE ablation, PVAC GOLD was reintroduced to assess PV isolation in SR. Ablation of the superior caval vein was performed in selected patients. The ablation catheters and phased RF generator are elaborated in Fig. 1.

### Data collection

Data was collected using a Research Electronic Data Capture (RedCap) system. Custom-built electronic case report forms were designed to capture the data for this registry, incorporating real-time data validation, for integrity checks and to facilitate audits to assure data quality. Detailed information on patient demographics, medical history, imaging, medication, procedural characteristics, procedural outcomes and safety outcomes were collected from hospital records for up until 3 months after the initial procedure.

### Study endpoints

The primary procedural outcome was acute success, defined as electrical isolation of all PVs. An assessment of entrance block for all targeted PVs was performed to confirm electrical isolation, additional pacing manoeuvres, exit block or provocative agents were performed at the discretion of the operator. Entrance block was defined as the absence or dissociation of electrical activity in the pulmonary veins, assessed using the PVAC GOLD catheter. PV touch-up using additional ablation catheters was classified as failure. Furthermore, data on procedure time, ablation time, fluoroscopy time, rhythm at the start of the procedure, PV anatomy, number of applications per vein, isolation per PV, number of additional ablations, cardioversions during the procedure and rhythm at the end of the procedure were acquired.

The primary safety outcome was the incidence of all major adverse events occurring up until 3 months after the ablation procedure, defined in compliance with the HRS/EHRA/ECAS/APHRS/SOLAECE expert consensus statement of 2017 [2]. Additionally, all non-major procedure–related adverse events were collected. Incidence and distribution of complications were compared in three ways: (1) paroxysmal AF versus non-paroxysmal AF, (2) PVI only versus PVI with additional substrate modification and (3) additional substrate modification with PVAC GOLD versus PVAC GOLD MASC MAAC. Finally, the number of complications with permanent sequelae was assessed and compared between groups. Complications with permanent sequelae were defined as those leading to death or causing harm not resolving within 12 months. All possible adverse events were reviewed by an independent adverse event committee. Vascular access events which did not require treatment or follow-up besides renewed or continued pressure bandage during non-prolonged hospitalization were not classified as an adverse event.

### Statistical analysis

Data is presented as mean and standard deviation for continuous variables, median and interquartile range for non-normally distributed variables and as numbers and percentages for categorical variables. Continuous data was compared using unpaired *t* test or Mann-Whitney *U* test when appropriate. Post hoc analysis was performed using Bonferroni. Categorical data was compared using *χ*^2^ test or Fishers exact test when a small number of events were observed. Primary outcomes were assessed using univariate and multivariable analyses using logistic regression. Variables were included when the *p* value was < 0.1. All tests were two-tailed and the limit for statistical significance was set at *p* < 0.05. Variables with ≥ 10% missing values were excluded. Statistical analysis was performed using SPSS for Windows software, version 24.0 (SPSS Inc., Chicago, IL, USA).

## Results

### Baseline characteristics

**Fig. 2:**
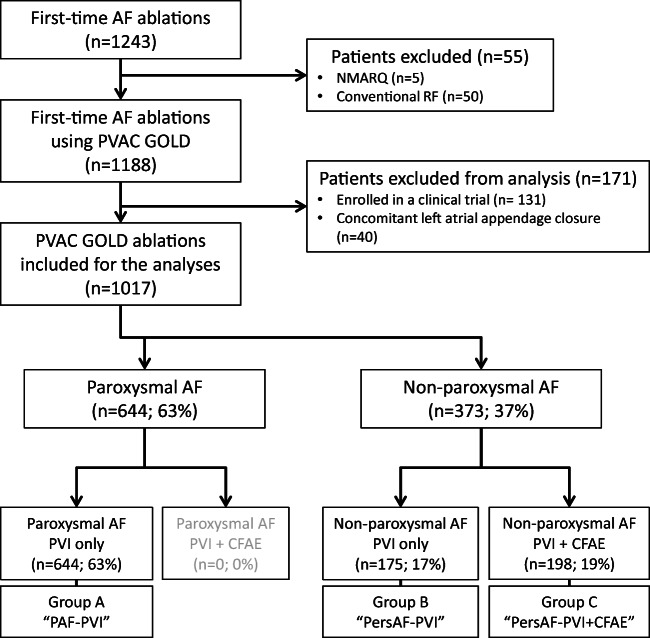
Flowchart illustrating enrollment and group allocation. AF, atrial fibrillation; RF, radiofrequency ablation

A total of 1017 patients were treated using PVAC GOLD and included for the analyses. Enrolment, inclusion for analyses and treatment group allocation are illustrated in Fig. 2. The baseline characteristics are shown in Table 1. Patients in the PAF-PVI group were more often female, were younger and had more favourable demographic characteristics and fewer comorbidities compared with the non-paroxysmal AF patients (all *p* < 0.05). Comparing patients within the non-paroxysmal AF groups, the PersAF-PVI + CFAE group was older (*p* = 0.041), had more often long-standing persistent AF (*p* = 0.004) and had more mitral valve regurgitation (*p* = 0.003). Ninety-eight percent of all patients had failed on class I or III anti arrhythmic drugs and no patients had undergone prior catheter ablation or surgical treatment for AF. Continued oral anticoagulation was used in 98% of patients, 59% VKA and 39% NOAC, with a higher percentage of NOAC use in the paroxysmal patients. The number of procedures per operator was equally distributed between groups.

**Table 1:** Baseline characteristics

**Demographics**	**PAF-PVI,** ***n*** **= 644**	**PersAF-PVI,** ***n*** **= 175**	**PersAF-PVI + CFAE,** ***n*** **= 198**	***p*** **value**
Gender male	66.6%	78.9%	78.3%	< 0.001
Age (years), mean ± SD	61.1 ± 10.5	61.0 ± 10.6	63.1 ± 9.3	0.047
BMI (kg/m^2^), mean ± SD	26.6 ± 4.0	27.4 ± 3.6	27.9 ± 4.5	< 0.001
Type of AF				
Paroxysmal	100%	0%	0%	-
Persistent	0%	95.4%	86.4%	0.003
Long-standing persistent	0%	4.6%	13.6%	0.003
CHA_2_DS_2_-VASC, mean ± SD	1.6 ± 1.3	1.8 ± 1.5	1.9 ± 1.6	0.011
HASBLED, median [iqr]	1 [0–2]	1 [0–2]	1 [0–2]	0.010
LAVI				
Normal < 34 ml/m^2^	67.8%	46.6%	39.1%	< 0.001
Slightly dilated 34–38 ml/m^2^	20.1%	33.5%	38.5%	< 0.001
Moderately dilated 42–48 ml/m^2^	6.2%	9.3%	9.9%	< 0.001
Severely dilated > 48 ml/m^2^	5.9%	10.6%	12.5%	< 0.001
Reduced LVEF < 50%	8.2%	21.9%	28.3%	< 0.001
Mitral valve regurgitation grade ≥ 2	7.2%	9.1%	16.2%	0.001
**Comorbidities**	**PAF-PVI**	**PersAF-PVI**	**PersAF-PVI + CFAE**	***p*** **value**
Congestive heart failure	4.3%	14.3%	13.6%	< 0.001
Hypertension	39.8%	46.3%	50.0%	0.024
Diabetes	5.9%	6.9%	9.1%	0.289
Stroke/TIA	4.7%	10.9%	11.6%	< 0.001
Vascular disease	16.3%	20.6%	14.6%	0.279
OSAS	4.8%	4.0%	7.6%	0.227
Reduced kidney function	7.9%	9.1%	12.3%	0.194
**Medication**	**PAF-PVI**	**PersAF-PVI**	**PersAF-PVI + CFAE**	***p*** **value**
OAC use at the time of ablation	97.5%	99.4%	99.5%	0.108
None	2.5%	0.57%	0.51%	< 0.001
VKA	54.2%	68.0%	67.7%	< 0.001
NOAC	43.3%	31.4%	31.8%	< 0.001

*BMI*, body mass index; *SD*, standard deviation; *iqr*, interquartile range; *AF*, atrial fibrillation, *LAVI*, left atrial volume indexed; *LVEF*, left ventricular ejection fraction; *TIA*, transient ischemic attack; *OSAS*, obstructive sleep apnoea syndrome; *OAC*, oral anticoagulant; *VKA*, vitamin K antagonist; *NOAC*, novel oral anticoagulant

### Acute success

High percentages of successful PVI could be achieved in all groups, 97.5%, and 3955 out of 3988 PVs (99.2%) could be successfully isolated, with comparable acute isolation rates between groups (*p* = 0.084 and *p* = 0.995) (Table 2). In 25 patients, acute success was not achieved. In 20 (2.0%) patients, despite sufficient contact and power, local PV potentials were still identified after ablation. In two patients (0.20%), the RIPV could not be reached to perform adequate ablation and in three patients (0.29%), the procedure had to be terminated prematurely before all PVs could be isolated or checked for entrance block due to worsened patient condition. Adjusted for baseline characteristics, no differences in acute success was found between groups (*p* = 0.108). The presence of an LCPV was associated with a slight but significant reduction in acute success (97.5% versus 95.3%, *p* = 0.030, OR 2.776, 95% CI 1.136–6.784).

**Table 2:** Procedural outcomes

**Procedure characteristics**	**PAF-PVI,** ***n*** **= 644 (%)**	**PersAF-PVI,** ***n*** **= 175 (%)**	**PersAF-PVI + CFAE,** ***n*** **= 198 (%)**	***p*** **value**
Sinus rhythm at start	89.3	79.4	15.2	< 0.001
‘Normal’ anatomy	83.4	85.7	81.3	0.522
LCPV	12.6	12.0	13.6	0.886
RMPV	5.3	2.3	6.6	0.147
ECVs performed	33.0	45.4	86.2	< 0.001
**Procedure outcomes**	**PAF-PVI (%)**	**PersAF-PVI (%)**	**PersAF-PVI + CFAE (%)**	***p*** **value**
PVs isolated	99.2	98.7	99.5	0.995
Acute success^a^	97.7	95.4	99.0	0.084
Remaining PV potentials	1.7	4.0	1.0	0.105
Anatomical limitation	0.31	0	0	1.000
Patient condition	0.31	0.57	0	0.512

^a^Acute success defined as electrical isolation of all pulmonary veins

‘*Normal*’ *anatomy*, 2 left and 2 right pulmonary veins; *LCPV*, left common pulmonary vein; *RMPV*, right middle pulmonary vein; *ECV*, electro cardioversion; *PV*, pulmonary vein; *PVI*, pulmonary vein isolation

### Procedural characteristics

**Fig. 3:**
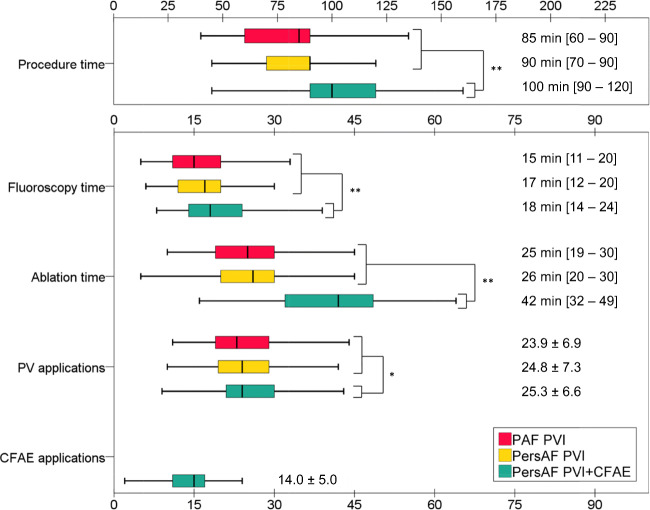
Procedure and ablation characteristics compared between groups. Box represents median and inter quartile range. The whiskers all other values. *p < 0.05; **p < 0.001. PV, pulmonary vein; CFAE, complex fractionated atrial electrogram

An overview of the procedural characteristics is summarized in Fig. 3 and presented in detail in supplementary Table S1. As expected, the total procedure, ablation and fluoroscopy times were all longer in the PersAF-PVI + CFAE group compared with the PAF-PVI and PersAF-PVI groups (all *p* < 0.001), but no difference between the PAF-PVI and PersAF-PVI groups (*p* = 0.306, *p* = 0.088, *p* = 0.233, respectively). In the PersAF-PVI + CFAE group, the weighted mean difference for the procedure time was 19 min longer (95% CI 14–22), the ablation time was 17 min longer (95% CI 14–19) and the fluoroscopy time was 3 min longer (95% CI 2–4) compared with the PVI-only groups. The mean total number of applications to achieve PVI was 24 ± 7 and numerically comparable between groups (Fig. 3). Furthermore, in the PersAF-PVI + CFAE group, 14 ± 5 CFAE applications were applied. Additional substrate modification was performed using the catheters MASC and MAAC in 154 patients (78%) and using the PVAC GOLD catheter in 44 patients (22%). CFAE applications were performed at the LA roof, the lateral wall between the LIPV and mitral annulus, and LA floor from lateral to septum with MAAC (8 ± 4 applications) and on the LA septum with the MASC (6 ± 2 applications). Ablation details, applications per group, applications per vein and secondary procedure outcomes are provided in supplementary Tables S1 and S2. Overall, 1000 patients (98%) could be timely discharged from the hospital on day 1 after the procedure and 9 (1%) on day 2 after the procedure. Complications (12/17) were the main reason for delayed discharge after 24 h. Delayed discharge did not differ between paroxysmal AF and non-paroxysmal AF (1.09% versus 2.95%, *p* = 0.271), between PVI only and PVI with substrate modification (1.59% versus 2.53%, *p* = 0.369) or between substrate modification using PVAC GOLD and PVAC GOLD + MASC + MAAC (3.2% versus 0.0%, *p* = 0.589).

### Safety outcomes

In 1017 procedures, a total of 44 complications were observed in 43 patients (4.23%): Major complications were seen in 19 (1.87%) and non-major procedure–related complications were seen in 25 (2.46%) patients. Complications leaving permanent sequelae were rare and occurred in only four (0.39%) patients. An overview of complications is illustrated in Table 3. Specifically, vascular access problems were among the more common complications, while a low rate of stroke/TIA (0.39%), tamponade (0.20%) and phrenic nerve paralysis (0.10%) was observed. Adjusted for baseline characteristics, all complications, major complications, non-major complications and permanent sequelae did not differ between groups (*p* = 0.369, *p* = 0.457, *p* = 0.199 and *p* = 0.465 respectively).

**Table 3:** Safety outcomes

Safety outcomes	PAF-PVI, *n* = 644 (%)	PersAF-PVI, *n* = 175 (%)	PVI + CFAE, *n* = 198 (%)	Adjusted, *p* value
Major complications	1.86	2.86	1.01	0.457
Major vascular access	0.78	0.57	0.0	
Bradycardia	0.16	0.57	1.01	
Tamponade	0.16	0.57	0.0	
Stroke^a^	0.31	0.0	0.0	
All-cause mortality^a^	0.16	0.0	0.0	
Phrenic nerve paralysis	0.16	0.0	0.0	
Major pericarditis	0.16	0.0	0.0	
Sedation related^a^	0.0	0.57	0.0	
Major bleeding	0.0	0.57	0.0	
Non-major complications	1.86	4.00	3.03	0.199
Transient ST elevation	0.47	1.14	1.52	
Minor bleeding	0.62	1.14	0.51	
Minor pericarditis	0.16	0.57	0.51	
Sedation related	0.31	0.0	0.51	
TIA	0.31	0.0	0.0%	
Minor vascular access	0.16	0.57	0.0%	
Complications overall	3.73	6.86	4.04	0.369

^a^Complications leaving permanent sequelae

Vascular access complications; divided in major: pseudoaneurysms, fistulae or any vascular access complication requiring intervention or transfusion, and minor: haematoma or leakage from the access site. *TIA* transient ischemic attack

Major complications with permanent sequelae were seen in four patients (0.39%). One immunocompromised patient was readmitted 3 days after the ablation procedure due to a severe pneumosepsis and died 5 days later. Autopsy confirmed a fulminant pneumonia, while no signs of bleeding, tamponade or fistula were seen. Although the patient underwent the procedure under conscious sedation, a relation to the hospital admission was deemed possible. Two patients presented with focal neurological deficit during admission. Both patients were treated conservatively and evaluated by a neurologist, showing a modified Rankin score of 1/6 (no significant disability) and 2/6 (slight disability) at 3 months. One sedation-related hypoxemia occurred in a patient with obstructive sleep apnoea syndrome, leading to cardiac arrest and admission to the intensive care unit for several days, with persisting myoclonic spasms after discharge. Complications leaving permanent sequelae did not differ between patients with paroxysmal AF and non-paroxysmal AF (0.37% versus 0.51%, *p* = 0.580), between PVI-only cases and those with additional substrate modification (0.49% versus 0.0%, *p* = 1.000) or between substrate modification using PVAC GOLD and PVAC GOLD + MASC + MAAC (0.0% versus 0.0%).

Major complications without permanent sequelae were observed in an additional 15 patients (1.47%). One early and one late cardiac tamponade requiring pericardiocentesis occurred. Both patients were successfully treated and could be discharged without symptoms. One phrenic nerve paralysis with symptoms persisting > 3 months but resolving within 1 year was seen. Six major vascular access complications occurred and one non-procedure-related bleeding requiring transfusion. Pericarditis requiring hospitalization was seen in one patient, which could be treated using oral medication. In four patients, bradycardia requiring pacing was seen. All showed sinus node dysfunction requiring pacing and were treated with a DDD pacemaker. No bradycardia due to AV block was seen. No myocardial infarction or atrial-oesophageal fistulae were seen. Major complications did not differ between patients with paroxysmal AF versus non-paroxysmal AF (1.86% versus 1.87%, *p* = 1.000), between PVI-only cases compared with those with additional substrate modification (2.08% versus 1.01%, *p* = 0.556) or between substrate modification using PVAC GOLD and PVAC GOLD + MASC + MAAC (1.3% versus 0.0%, *p* = 1.000).

Non-major complications occurred in 25 patients (2.46%). Minor vascular access complications consisted of haematomas and minor groin bleeds which occurred in nine patients (0.88%). Vascular complications were equally distributed among all groups (*p* = 0.226). Eight patients showed peri-procedural transient ST elevation (0.79%), which all resolved spontaneously within 5 min leaving no permanent changes on the electrocardiogram or alterations in left ventricular function in any of the patients. These ST elevations were all adjudicated to be due to air bubbles after flushing of the sheath. Non-major complications did not differ between patients with paroxysmal AF versus non-paroxysmal AF (1.86% versus 3.49%, *p* = 0.140), between PVI-only cases compared with those with additional substrate modification (1.95% versus 3.03%, *p* = 0.411) or between substrate modification using PVAC GOLD and PVAC GOLD + MASC + MAAC (3.9% versus 0.0%, *p* = 0.341).

## Discussion

### Main findings

This is the first and only single-centre, multi-operator large real-world registry evaluating procedural outcomes and safety using the second-generation PVAC GOLD in over 1000 patients. The present study shows a high rate of acute successful PVI, while retaining swift procedure times and a desirable safety profile for paroxysmal, persistent and long-standing persistent AF patients. Furthermore, additional CFAE ablations in the left atrium with either PVAC GOLD or MASC and MAAC did not affect the safety outcomes.

### Acute success

Acute success was attained in 97.5% of patients and 99% of PVs, similar to isolation rates observed with the first-generation PVAC [5–7]. These high rates of procedural success are consistent with clinical trials investigating the latest-generation cryoballoon or conventional RF ablation, showing acute success rates of 97 up to 100% of cases [8–11]. Large registries and multinational surveys assessing irrigated RF and cryoballoon ablation resembling a more real-world population and show lower success rates (83–95%) [12–14]. Therefore, the results we found are reassuring and confirm procedural efficacy for paroxysmal and persistent AF patients. Durable PVI and long-term arrhythmia-free survival was not assessed in this paper and further research is needed to address these topics.

### Procedural characteristics

The PVAC GOLD system encompasses a simple and efficacious technique to achieve PVI, leading to favourable procedural characteristics. The median procedure time was less than 90 min in the PVI-only groups and 100 min in the PersAF-PVI + CFAE group. Likewise, fluoroscopy time and ablation time were short, confirming the swift procedural characteristics previously reported in smaller trials and registries [6, 7, 15]. Compared with the reported procedural characteristics of conventional point-by-point ablation [9, 10, 12, 13, 16] and to a lesser extent to cryoballoon ablation [8, 10, 11, 13], we found PVAC GOLD to have considerably shorter procedure times, fluoroscopy times and ablation times. These findings are in agreement with a meta-analysis performed by Kabunga et al., who compared phased RF, irrigated RF and cryoballoon [17].

### Safety outcomes

Safety remains an important topic in AF ablation. Although regularly reported, heterogeneity in screening methods and the definitions used to include and classify complications remains problematic. Reported major safety outcomes widely vary between 1.4 and 16% and focus mainly on paroxysmal AF [6–8, 13, 15, 16, 18]. We reported outcomes according to the 2017 consensus statement to support uniform data presentation. Procedure-related complications were seen in 4.2% of patients and major complications were seen in 1.87% of patients, of which only 0.39% left sequelae. Although 3 months of follow-up was assessed to determine procedural safety outcomes, late-onset or less clinically overt complications might be underreported in this registry.

We found no differences in safety outcomes between patients with paroxysmal and non-paroxysmal AF. Furthermore, additional CFAE ablations with PVAC GOLD or MASC and MAAC in the left atrium did not impact safety outcomes. Previous, mostly small studies on the second-generation PVAC GOLD catheter reported major complications ranging from 0 to 6.5% [6–8, 15]. More recent and larger studies by Spitzer et al. and De Greef et al. showed more comparable major complication rates below 2% [15, 18].

Compared with important landmark trials like FIRE and ICE, CABANA and STAR-AF [9, 10, 19], we observed lower rates of major complications. A large Italian registry including 2167 patients from 29 centres using open-irrigated catheters showed similar complication rates of 3.7% and 0.20% complications with permanent sequelae [16]. Similar results were found in a German registry for paroxysmal AF, including 3775 patients who underwent cryoballoon or RF ablation and reported 4.6% major adverse events in both cryoballoon and RF ablation [8].

Major complication rates reported on PVAC GOLD seem to be on the lower end of the spectrum. An explanation might be the low incidence of phrenic nerve palsy and cardiac tamponade.

In contrast to cryoballoon ablation procedures, phrenic nerve palsy is rare and occurred in only a single patient. Of interest, when ablation is performed and the phrenic nerve is near, typically capture of the phrenic with rapid oscillation of the right hemi-diaphragm is both visible and tangible for the operator. This early warning sign allowing immediate discontinuation of energy delivery, as well as the awareness to stay outside the tubular part of the PV may be an explanation for the low rate of phrenic nerve palsies.

Compared with single-shot devices, conventional point-by-point RF ablation may cause more cardiac tamponades and pericardial effusions [7, 8, 10, 18]. The over-the-wire design and the use of a circular array might help distribute pressure over a larger area, thereby preventing stress on small areas and risk of steam pops and perforations as is evident from the very low number of tamponade which were usually related to the transseptal puncture.

Using PVAC GOLD, we observed a low stroke/TIA rate (0.39%), which is in line with or even lower than in other large AF ablation trials such as STAR-AF (0.53%), FIRE and ICE (0.5% radiofrequency, 0.5% cryoballoon) and CABANA (0.3%) and a smaller single-centre report by Spitzer et al. on 384 patients treated using PVAC GOLD (0.52%) [9, 10, 15, 19]. This is reassuring in the sense that using multi-electrode catheters with non-irrigated phased RF energy appears to be as safe as other technologies and does not lead to clinical neurological sequelae. Other factors such as our strategy of uninterrupted anticoagulation have also been shown to reduce the occurrence TIA and stroke, even though it would not be effective against the formation of thermally induced coagulum and subsequent stroke, TIA or ACE. The main reasons for the design change of PVAC platinum with 10 electrodes to PVAC GOLD with 9 electrodes however was the concern for a higher asymptomatic cerebral embolism rate [3, 4]. Extensive studies in animal models showed that it was the interaction between electrodes 1 and 10 on the catheter that led to overheating with gas and particle formation, which caused temporary micro-emboli in the brain that completely resolved shortly after the procedure without causing any neurological deficit. Avoiding simultaneous ablation on electrodes 1 and 10 resolved the ACE problems (ERASE trial), which led to the design of a 9-electrode array with gold electrodes for better heat dissipation with a confirmed extremely low ACE rate (PRECISION GOLD trial). Given these reassuring studies that were in part done at our own centre, DW-MRI was not part of our regular peri-procedural assessment of safety as it is a burden to patients, as well as a logistical challenge that does not seem cost-effective as it has no consequence for the patient. The issue of ACE lesions and their clinical significance after any type of ablation however remains in part unresolved, as is the fact that some operators achieve low rates (PRECISION GOLD De Greef et al. 2.1% multicentre trial with strict procedural guidance, core lab strict MRI assessment) while others do not (Keçe et al., 23%, single-centre experience) [3, 20].

### CFAE ablations

CFAE ablations were safely performed in half of the patients with persistent AF and in 77% of the patients with long-standing persistent AF. CFAE ablations did significantly lengthen the procedure and required additional fluoroscopy time, but the major complication rate and non-major complication rate were still low and equal to the PVI-only groups. This was also reported by Chen et al., who performed a meta-analysis on efficacy and safety of adjunctive substrate modification, as well as results found in the STAR-AF trial [9, 21]. Despite a safe and successful procedure, the effect of CFAE ablations as a first-line treatment for atrial fibrillation on arrhythmia-free survival remains controversial [9].

### Limitations

The current study was designed to present daily practice data on acute success and safety in different patient groups and treatment strategies using multi-electrode phased RF ablation. The choice for PVAC GOLD was not randomized and additional substrate modification was performed at the discretion of the operator. During the inclusion period, 96% of first-time AF ablations were performed using PVAC GOLD, depicting a true all-comers population. Consequently, no suitable control group with comparable standard of care and follow-up could be selected. Head-to-head comparison between the different groups must be interpreted with caution, as the patients within groups are not interchangeable and encompass different risk factors. Despite strong differences in patient characteristics, all groups showed similar and reassuring acute success rates and a low number of safety outcomes. Multicentre randomized clinical trials are the gold standard for comparison, but due to the low rate of incomplete PVI and safety outcomes as well as the rapid evolution of available techniques and alterations in standard of care, such trials will be unfeasible. Therefore, uniform reporting in large registries are of important value to appreciate ablation technologies. In our registry, no variables showed > 5% missing values and missings appeared to be at random. The primary outcome variables did not contain any missing values. While acute success rates are reassuring, outcomes on long-term arrhythmia-free survival are warranted.

## Conclusions

In conclusion, this study provides extensive data on the use of PVAC GOLD in a large, broad everyday practice population-based cohort. The second-generation multi-electrode phased radiofrequency pulmonary vein ablation catheter achieves successful circumferential ablation of the pulmonary veins, while reducing procedure times, for patients with paroxysmal, persistent and long-standing persistent AF. A desirable safety profile was found in all groups and was unaffected by additional CFAE ablations in the left atrium.

## Electronic supplementary material

ESM 1 (DOCX 20 kb)

## Abbreviations

ACE: Asymptomatic cerebral embolism
AF: Atrial fibrillation
CFAE: Complex fractionated atrial electrogram
LCPV: Left common pulmonary vein
LIPV: Left inferior pulmonary vein
LSPV: Left superior pulmonary vein
MAAC: Multi-array ablation catheter
MASC: Multi-array ablation catheter
NOAC: Non–vitamin K oral anticoagulation
PAF: Paroxysmal atrial fibrillation
PersAF: Persistent atrial fibrillation
PV: Pulmonary vein
PVAC: Pulmonary vein ablation catheter
PVI: Pulmonary vein isolation
RF: Radiofrequency
RIPV: Right inferior pulmonary vein
RMPV: Right middle pulmonary vein
RSPV: Right superior pulmonary vein
TIA: Transient ischemic attack
VKA: Vitamin K antagonist
