# Supplementary material for: Acute success and safety of the second-generation PVAC GOLD phased RF ablation catheter for atrial fibrillation
Source: J Interv Card Electrophysiol. 2020 Apr 6;60(2):261–70. doi: 10.1007/s10840-020-00728-8 (PMC7925470; doi:10.1007/s10840-020-00728-8)
Supplement: Supplementary file 1 — (DOCX 20 kb) [file 10840_2020_728_MOESM1_ESM.docx]

**Acute success and safety of the second generation PVAC GOLD phased RF ablation catheter for atrial fibrillation: results from the St. Antonius registry.**

Klaver M.N. MD^1^, Wintgens L.I.S.MD^1^, Wijffels M.C.E.F.MD, PhD^1^, Balt J.C. MD, PhD^1^, van Dijk V.F. MD^1^, Alipour A. MD, PhD^2^, Chaldoupi S.M. MD, PhD^3^, Derksen R. MD^4^, Boersma L.V.A. MD, PhD, FESC^1,5^

(1) Department of Cardiology, St. Antonius Hospital, Nieuwegein, The Netherlands

(2) Department of Cardiology, Rivierenland Hospital, Tiel, The Netherlands

(3) Department of Cardiology, Maastricht University Medical Centre, Maastricht, The Netherlands

(4) Department of Cardiology, Rijnstate Hospital, Arnhem, The Netherlands

(5) Department of Cardiology, Amsterdam University Medical Centres, location Academic Medical Centre, Amsterdam, The Netherlands

**Corresponding address**

St. Antonius ziekenhuis, R&D Cardiologie E1

t.a.v. M.N. Klaver

Koekoekslaan 1

3435CM Nieuwegein

The Netherlands

Telephone: 0031883200900

Fax: 0031306034420

E-mail: m.klaver@antoniusziekenhuis.nl

**Journal of intercentional cardiac electrophysiology**

**Supplementary tables**

| **Supplementary table S1** |  |  |  |  |  |
| --- | --- | --- | --- | --- | --- |
| **Procedure characteristics** | **Overall (n = 1017)** | **PAF PVI (n = 644)** | **PersAF PVI (n = 175)** | **PVI + CFAE**  **(n = 198)** | **p-value** |
| Sinus rhythm at start | **744 (73.2%)** | 575 (89.3%) | 139 (79.4%) | 30 (15.2%) | <0.001 |
| Procedure time (min) | **90 [70 – 100]** | 85 [60 – 90] | 90 [70 – 90] | 100 [90 – 120] | <0.001 |
| Ablation time (min) | **27 [21 – 34]** | 25 [19 – 30] | 26 [20 – 30] | 42 [32 – 48.5] | <0.001 |
| Fluoroscopy time (min) | **16 [12 – 21]** | 15 [11 – 20] | 17 [12 – 20] | 18 [14 – 24] | <0.001 |
| ECVs performed during procedure | **458 (45.5%)** | 210 (33.0%) | 79 (45.4%) | 169 (86.2%) | <0.001 |
| **Anatomy** | **Overall (n = 1017)** | **PAF PVI (n = 644)** | **PersAF PVI (n = 175)** | **PVI + CFAE**  **(n = 198)** | **p-value** |
| ‘Normal’ anatomy (2 LPVs 2 RPVs) | **848 (83.4%)** | 537 (83.4%) | 150 (85.7%) | 161 (81.3%) | 0.522 |
| LCPV | **129 (12.7%)** | 81 (12.6%) | 21 (12.0%) | 27 (13.6%) | 0.886 |
| RMPV | **51 (5.0%)** | 34 (5.3%) | 4 (2.3%) | 13 (6.6%) | 0.147 |
| **Ablations** | **Overall (n = 1017)** | **PAF PVI (n = 644)** | **PersAF PVI (n = 175)** | **PVI + CFAE**  **(n = 198)** | **p-value** |
| Total applications PVs | **24.33±6.95** | 23.92±6.90 | 24.78±7.36 | 25.28±6.64 | 0.037 |
| LSPV | **7.28±2.85** | 7.18±2.84 | 7.21±2.88 | 7.70±2.84 | 0.108 |
| LIPV | **6.24±2.87** | 6.13±2.80 | 6.60±3.02 | 6.27±2.96 | 0.200 |
| LCPV | **10.46±4.00** | 10.16±3.95 | 11.48±4.48 | 10.52±3.78 | 0.412 |
| RSPV | **5.39±2.45** | 5.39±2.51 | 5.23±2.21 | 5.52±2.45 | 0.537 |
| RIPV | **5.69±2.64** | 5.47±2.63 | 5.98±2.92 | 6.14±2.32 | 0.002 |
| RMPV | **2.58±1.92** | 2.62±1.83 | 4.00±3.67 | 1.92±0.86 | 0.118 |
| CFAE ablations | **14.06±4.96** | - | - | 14.06±4.96 | - |
| MASC | **5.94±2.36** | - | - | 5.94±2.36 | - |
| MAAC | **8.07±3.74** | - | - | 8.07±3.74 | - |
| ECV, electro cardioversion; LPV, left pulmonary veins; RPV, right pulmonary veins; LCPV, left common pulmonary vein; RMPV, right middle pulmonary vein; PV, pulmonary vein; LSPV, left superior pulmonary vein; LIPV, left inferior pulmonary vein; RSPV, right superior pulmonary vein; RIPV, right inferior pulmonary vein; CFAE, complex fractional atrial electrogram; MASC, Multi-array septal catheter; MAAC, multi-array ablation catheter. | | | | | |

| **Supplementary table S2** | | | | | |  |
| --- | --- | --- | --- | --- | --- | --- |
| **Procedure outcomes** | **Overall (n = 1017)** | **PAF PVI (n = 644)** | **PersAF PVI (n = 175)** | **PVI + CFAE**  **(n = 198)** | **p-value** | **Adjusted p-value** |
| Complete PVI | **992 (97.5%)** | 629 (97.7%) | 167 (95.4%) | 196 (99.0%) | 0.084 | 0.108 |
| PVs isolated | **3955/3988 (99.17%)** | 2508/2528 (99.21%) | 673/682 (98.68%) | 774/778 (99.49%) | 0.995 |  |
| Reasons for unsuccessful PVI |  |  |  |  |  |  |
| Remaining PV potentials | **20 (1.97%)** | 11 (1.71%) | 7 (4.00%) | 2 (1.01%) | 0.105 |  |
| Anatomical limitation | **2 (0.20%)** | 2 (0.31%) | 0 (0.0%) | 0 (0.0%) | 1.000 |  |
| Patient condition | **3 (0.29%)** | 2 (0.31%) | 1 (0.57 %) | 0 (0.0%) | 0.512 |  |
| Sinus rhythm at closure | **996 (97.9%)** | 637 (98.9%) | 171 (97.7%) | 188 (94.9%) | 0.003 |  |
| Length of stay (days) | **1.06±0.95 1 (1 – 29)** | 1.02±0.267 1 (1 – 6) | 1.20±13 1 (1 – 29) | 1.08±0.599 1 (1 – 7) | 0.083 |  |
| Discharge at day 1 | **1000 (98.3%)** | 638 (99.1%) | 169 (96.6%) | 193 (97.5%) | 0.031 |  |
| Discharge at day 2 | **1009 (99.2%)** | 641 (99.5%) | 174 (99.4%) | 195 (98.5%) | 0.278 |  |
| PVI, pulmonary vein isolation; PV, pulmonary vein; | | | | | |  |

| **Supplementary table S3** |  |  |  |  |  |  |
| --- | --- | --- | --- | --- | --- | --- |
| **Safety outcomes** | **Overall (n = 1017)** | **PAF PVI (n = 644)** | **PersAF PVI (n = 175)** | **PVI + CFAE**  **(n = 198)** | **p-value** | **Adjusted p-value** |
| **Major complications** | **19 (1.87%)** | **12 (1.86%)** | **5 (2.86%)** | **2 (1.06%)** | **0.444** | **0.438** |
| Major vascular access | 6 (0.59%) | 5 (0.78%) | 1 (0.57%) | 0 (0.0%) | 0.719 |  |
| Bradycardia | 4 (0.39%) | 1 (0.16%) | 1 (0.57%) | 2 (1.01%) | 0.143 |  |
| Tamponade | 2 (0.20%) | 1 (0.16%) | 1 (0.57%) | 0 (0.0%) | 0.352 |  |
| Stroke^a^ | 2 (0.20%) | 2 (0.31%) | 0 (0.0%) | 0 (0.0%) | 1.000 |  |
| All-cause mortality^a^ | 1 (0.10%) | 1 (0.16%) | 0 (0.0%) | 0 (0.0%) | 1.000 |  |
| Phrenic nerve paralysis | 1 (0.10%) | 1 (0.16%) | 0 (0.0%) | 0 (0.0%) | 1.000 |  |
| Major pericarditis | 1 (0.10%) | 1 (0.16%) | 0 (0.0%) | 0 (0.0%) | 1.000 |  |
| Sedation related^a^ | 1 (0.10%) | 0 (0.0%) | 1 (0.57%) | 0 (0.0%) | 0.172 |  |
| Major bleeding | 1 (0.10%) | 0 (0.0%) | 1 (0.57%) | 0 (0.0%) | 0.172 |  |
| **Non-major procedure related complications** | **25 (2.46%)** | **12 (1.86%)** | **7 (4.00%)** | **6 (3.03%)** | **0.205** | **0.240** |
| Transient ST elevation | 8 (0.79%) | 3 (0.47%) | 2 (1.14%) | 3 (1.52%) | 0.206 |  |
| Minor bleeding | 7 (0.69%) | 4 (0.62%) | 2 (1.14%) | 1 (0.51%) | 0.745 |  |
| Minor pericarditis | 3 (0.29%) | 1 (0.16%) | 1 (0.57%) | 1 (0.51%) | 0.305 |  |
| Sedation related | 3 (0.29%) | 2 (0.31%) | 0 (0.0%) | 1 (0.51%) | 0.747 |  |
| TIA | 2 (0.20%) | 2 (0.31%) | 0 (0.0%) | 0 (0.0%) | 1.000 |  |
| Minor vascular access | 2 (0.20%) | 1 (0.16%) | 1 (0.57%) | 0 (0.0%) | 0.352 |  |
| **Complications overall** | **44 (4.32%)** | **24 (3.73%)** | **12 (6.86%)** | **8 (4.04%)** | **0.190** | **0.199** |
| ^a^ complications leaving permanent sequelae  Vascular access complications; divided in minor: hematoma or leakage from the access site, major: pseudoaneurysms, fistulae or any vascular access complication requiring intervention or transfusion. TIA: transient ischemic attack | | | | | |  |
